# CTCF and Cohesin^SA-1^ Mark Active Promoters and Boundaries of Repressive Chromatin Domains in Primary Human Erythroid Cells

**DOI:** 10.1371/journal.pone.0155378

**Published:** 2016-05-24

**Authors:** Laurie A. Steiner, Vincent Schulz, Yelena Makismova, Kimberly Lezon-Geyda, Patrick G. Gallagher

**Affiliations:** 1 Department of Pediatrics, University of Rochester, Rochester, New York, United States of America; 2 Department of Pediatrics, Yale University School of Medicine, New Haven, Connecticut, United States of America; 3 Departments of Pathology and Genetics, Yale University School of Medicine, New Haven, Connecticut, United States of America; Osaka University, JAPAN

## Abstract

**Background:**

CTCF and cohesin^SA-1^ are regulatory proteins involved in a number of critical cellular processes including transcription, maintenance of chromatin domain architecture, and insulator function. To assess changes in the CTCF and cohesin^SA-1^ interactomes during erythropoiesis, chromatin immunoprecipitation coupled with high throughput sequencing and mRNA transcriptome analyses via RNA-seq were performed in primary human hematopoietic stem and progenitor cells (HSPC) and primary human erythroid cells from single donors.

**Results:**

Sites of CTCF and cohesin^SA-1^ co-occupancy were enriched in gene promoters in HSPC and erythroid cells compared to single CTCF or cohesin sites. Cell type-specific CTCF sites in erythroid cells were linked to highly expressed genes, with the opposite pattern observed in HSPCs. Chromatin domains were identified by ChIP-seq with antibodies against trimethylated lysine 27 histone H3, a modification associated with repressive chromatin. Repressive chromatin domains increased in both number and size during hematopoiesis, with many more repressive domains in erythroid cells than HSPCs. CTCF and cohesin^SA-1^ marked the boundaries of these repressive chromatin domains in a cell-type specific manner.

**Conclusion:**

These genome wide data, changes in sites of protein occupancy, chromatin architecture, and related gene expression, support the hypothesis that CTCF and cohesin^SA-1^ have multiple roles in the regulation of gene expression during erythropoiesis including transcriptional regulation at gene promoters and maintenance of chromatin architecture. These data from primary human erythroid cells provide a resource for studies of normal and perturbed erythropoiesis.

## Introduction

The dynamic interplay between DNA methylation, histone modification, and chromatin structure are critical for establishing and maintaining appropriate patterns of mammalian gene expression. In vertebrates, the highly conserved, multifunctional CCTC-binding factor CTCF binds throughout the genome in a sequence-[1] and DNA methylation-specific manner. [2–4] CTCF has multiple functions including acting directly at gene promoters to regulate transcription, mediating long-range chromatin interactions, and it is the best characterized chromatin domain insulator-associated protein in vertebrates.

The cohesin complex plays numerous roles in mammalian gene regulation including promoting transcription factor binding at enhancers [5, 6] and promoting cell-type specific gene activation by facilitating DNA-promoter interactions through cell-type specific DNA-looping.[7, 8] CTCF may co-localize with cohesin [9–13] which then targets both proteins to specific sites in the genome. Interactions between the cohesin complex and CTCF mediate cell-type specific long-range chromatin contacts and modulate the enhancer-blocker activity of CTCF.[14–16] The cohesin complex is composed of four proteins Smc1, Smc3, Scc1, and either SA-1 or SA-2.[17] SA-1 and SA-2 are closely related homologs of Scc3, whose presence in cohesin complexes is mutually exclusive, leading to two highly related, but distinct complexes, cohesin^SA-1^ and cohesin.^SA-2^ [18, 19] The SA-1 component of the cohesin complex has been shown to directly interact with CTCF, mediating many of the above functions.[9]

The goal of these studies was to gain insight into the roles of CTCF, cohesin^SA-1^, and their association with gene expression and chromatin domain organization in erythroid development. Chromatin immunoprecipitation coupled with high throughput sequencing and mRNA transcriptome analyses via RNA-seq were performed in primary human hematopoietic stem and progenitor cells (HSPC) and primary human erythroid cells from single donors. Changes in sites of CTCF and cohesin^SA-1^ occupancy and their association with gene expression were observed. Cell type-specific CTCF sites in erythroid cells were linked to highly expressed genes. Repressive chromatin domains increased in both number and size during hematopoiesis, with many more repressive domains in erythroid cells than HSPCs. CTCF and cohesin^SA-1^ marked the boundaries of these repressive chromatin domains in a cell-type specific manner. These genomic data support the hypothesis that CTCF and cohesin^SA-1^ have multiple roles in the regulation of gene expression during erythropoiesis including transcriptional regulation at gene promoters and maintenance of chromatin architecture.

## Methods

### Cell selection and RNA analyses

Human CD34+-selected hematopoietic stem and progenitor cells (hereafter called HSPCs) isolated at >95% purity were obtained from the Yale Cooperative Center for Excellence in Molecular Hematology from unused clinical specimens. Erythroid progenitor cells were cultured and isolated as described.[20] Immunomagnetic bead selection was used to select a population of cells based on expression of CD71 (transferrin receptor) and CD235a (glycophorin A), representing the R3/R4 cell population of nucleated erythroid cells defined by Zhang *et al*.[21] at >95% purity as assessed by analytic FACS (Figure A in S1 File).

To avoid donor-to-donor variability observed in hematopoietic cells, including differences in age, gender, genetic background, etc., [22–24] studies, i.e. RNA-seq and ChIP-seq of CTCF and cohesin^SA1^, were performed using CD34+ and erythroid cells derived from the same donor.

RNA was isolated and prepared for RNA-seq analyses as described.[20] Samples were sequenced on an Illumina HiSeq 2000 using 76bp-single end reads. FASTQ format sequencing reads were aligned to the hg19 genome, NCBI Build 37, using TopHat Version 2.0.4 software with default parameters except minimum anchor length of 12. The EdgeR program was used to identify differences in expression of RefSeq transcripts. Filtering included transcripts with >1 tag/million reads in 3 or more samples.

### Chromatin immunoprecipitation and high throughput sequencing

ChIP assays were performed as previously described.[20, 25, 26] Samples were immunoprecipitated with antibody against CTCF (Creative Diagnostics, DMABT-H19813), the SA-1 subunit of cohesin (Abcam ab4457), trimethyl histone H3 lysine 27 (Abcam ab6002) or nonspecific rabbit IgG (sc-2091 Santa Cruz). DNA processing and high throughput sequencing were performed as described.[20] Because of the age, gender, and genetic background differences noted above, and the growing realization genetic variability influences epigenetic findings, [27] parallel RNA-seq and ChIP-seq of CTCF and cohesin^SA1^ data sets from individual donors were analyzed together.

### Analyses of ChIP-seq results

The MACS program version 1.4.0rc2 was used to identify peaks with a p-value<10e-5 and a fold enrichment >6 for erythroid SA1 and >8 for the other samples.[28] Quality control analyses of Chip-seq data were performed using Picard MarkDuplicates (http://broadinstitute.github.io/picard), Phantompeakqualtools and the DiffBind package.[29, 30] The DiffBind analysis used fold-change filtered peaks with defaults parameters (minOverlap = 2). The best replicate for each condition was chosen for further analysis. Localization of CTCF and cohesin^SA1^ binding sites relative to known genes was done using the BEDTools software package.[31] Comparison of CTCF genome-wide binding data sets generated through the Broad Institute as part of the ENCODE consortium were acquired through the UCSC Genome Browser (http://genome.ucsc.edu/). Motif finding was done using the Homer software package.[32] Motifs discovered by Homer were compared against the Homer database of known motifs from TRANSFAC, JASPAR and public ChIP-seq data.[33] The Genomic Regions Enrichment Annotations Tool (GREAT) was used to analyze functional significance of *cis*-regulatory regions identified by ChIP-seq.[34] Broad regions of H3K27me3 binding were identified using SICER.[35] Regions with >3 fold enrichment were merged with neighboring regions within 2000 bases, and the resulting regions larger than 2000 bases were used for H3K27me3 domain analysis. Co-localization *p*-values were obtained by randomization of genomic intervals within the human genome excluding gap regions for 1000 iterations.

### Validation of ChIP-seq results

Primers were designed for representative binding regions for both CTCF and cohesin^SA-1^ in the target genes identified by the MACS program (Table A in S2 File). Immunoprecipitated DNA was analyzed by quantitative real-time PCR as described.[25] All quantitative ChIP validation experiments were performed at least in triplicate.

### Data access

The raw data files generated by RNA-seq and ChIP-seq analyses have been submitted to Gene Expression Omnibus (GEO, http://www.ncbi.nlm.nih.gov/geo/ Reference series number GSE67893).

## Results

### CTCF and cohesin^SA-1^ ChIP-seq and mRNA expression analyses in human hematopoietic stem and progenitor (HSPC) and primary erythroid cells

ChIP-seq was performed utilizing antibodies specific for CTCF and the SA-1 component of the cohesin complex (cohesin^SA-1^) to generate genome-wide maps of CTCF and cohesin^SA-1^ binding in primary human HSPC and erythroid cell chromatin. Quality control analyses of Chip-seq data for read duplication, strand cross correlation, and principal components clustering demonstrated the data were of high quality (Table B in S2 File and Figure B-D in S1 File). Validation of CTCF and cohesin^SA-1^ enrichment at selected peaks was performed by quantitative ChIP PCR (Figure C in S1 File and Tables C and D in S2 File). In the replicate chosen for analyses, the MACS program identified 50,798 sites of CTCF and 42,072 sites of cohesin^SA-1^ occupancy in HSPC cell chromatin and 49,417 sites of CTCF and 40,511 sites of cohesin^SA-1^ occupancy in erythroid cell chromatin (p<10e-5)(Table E in S2 File).

Transcriptome analyses were performed using mRNA isolated from human HSPC and erythroid cells using RNA-seq. In HSPC cells, 13,106 transcripts were detected (median count per million reads >1), while in erythroid cells 12,790 transcripts were detected. Five thousand two hundred thirty two transcripts were differentially expressed by more than 2 fold between HSPC and erythroid cells, with 2289 genes up regulated in erythroid cells and 2943 down regulated in erythroid cells.

### Sites of CTCF and cohesin^SA-1^ co-occupancy are enriched in gene promoters

Overlap of sites of CTCF and cohesin^SA-1^ occupancy were analyzed in HSPC and erythroid cells (Figure E in S1 File). In erythroid cells, more CTCF sites were co-occupied with cohesin^SA-1^ than CTCF sites lacking cohesin^SA-1^ (co-occupied: 26,658 vs. CTCF alone 22,869). In contrast, in HSPCs, the majority of CTCF sites lacked cohesin^SA-1^ co-occupancy (co-occupied: 18,179 vs CTCF alone: 29,000).

**Fig 1:**
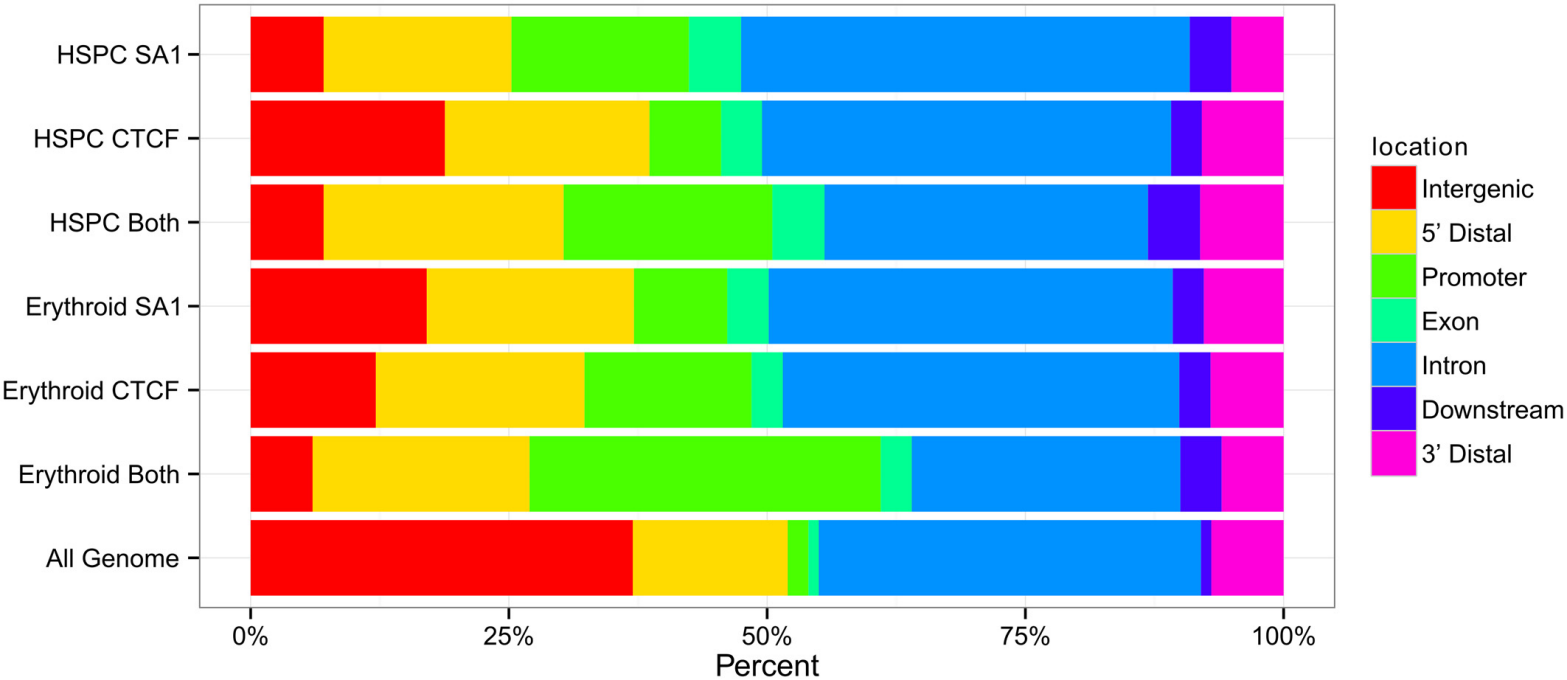
Distribution of CTCF and cohesin^SA-1^ occupancy and co-occupancy in human primary hematopoietic stem and progenitor cell (HSPC) and primary erythroid cell chromatin. ChIP-seq was performed with antibodies against CTCF and cohesin^SA-1^ in human primary hematopoietic stem and progenitor cell (HSPC) and primary erythroid cell chromatin. Sites of protein occupancy were determined by MACS. The human genome was portioned into seven bins relative to known genomic features associated with RefSeq genes. The percentage of the human genome represented by each bin was color coded, and the distribution of peaks of CTCF and cohesin^SA-1^ in each bin graphed on the color coded bar. Abbreviations: TSS: transcriptional start site; TES: transcriptional end site. Intergenic: >50Kb from a gene. 5’ distal 1-50Kb upstream of TSS. Promoter: within 1Kb of TSS. Downstream: within 1 Kb of TES. 3’ Distal 1-50Kb downstream of TES.

In both HSPC and erythroid cell chromatin, CTCF and cohesin^SA-1^ binding sites were enriched in 5’ flanking regions and promoter regions, and, intergenic regions were underrepresented relative to genome composition (Fig 1). In both cell types, sites of CTCF and cohesin^SA-1^ co-occupancy were increased at gene promoters compared to singly occupied sites at gene promoters, 20% in HSPC cells and 31% in erythroid cells.

The Homer algorithm was utilized to identify over represented DNA motifs at sites of CTCF and cohesin^SA-1^ binding. In HSPC cell chromatin, the most common motif identified at co-occupied peaks and CTCF peaks without cohesin^SA-1^ was nearly identical to the CTCF consensus motif identified by Kim *et al*. in primary human fibroblasts (Figure F in S1 File).[1] The most common motif identified at cohesin^SA-1^ binding sites in HSPC cell chromatin was a BRCA1-binding motif. In erythroid cell chromatin, the most common motif identified at co-occupied peaks and CTCF peaks without cohesin^SA-1^ was CTCF, while the most common motif identified at cohesin^SA-1^ binding sites without CTCF was Sp1. Other over represented motifs are shown in Figure G in S1 File.

### A subset of CTCF sites are cell-type specific in HSPC and erythroid cell chromatin

**Fig 2:**
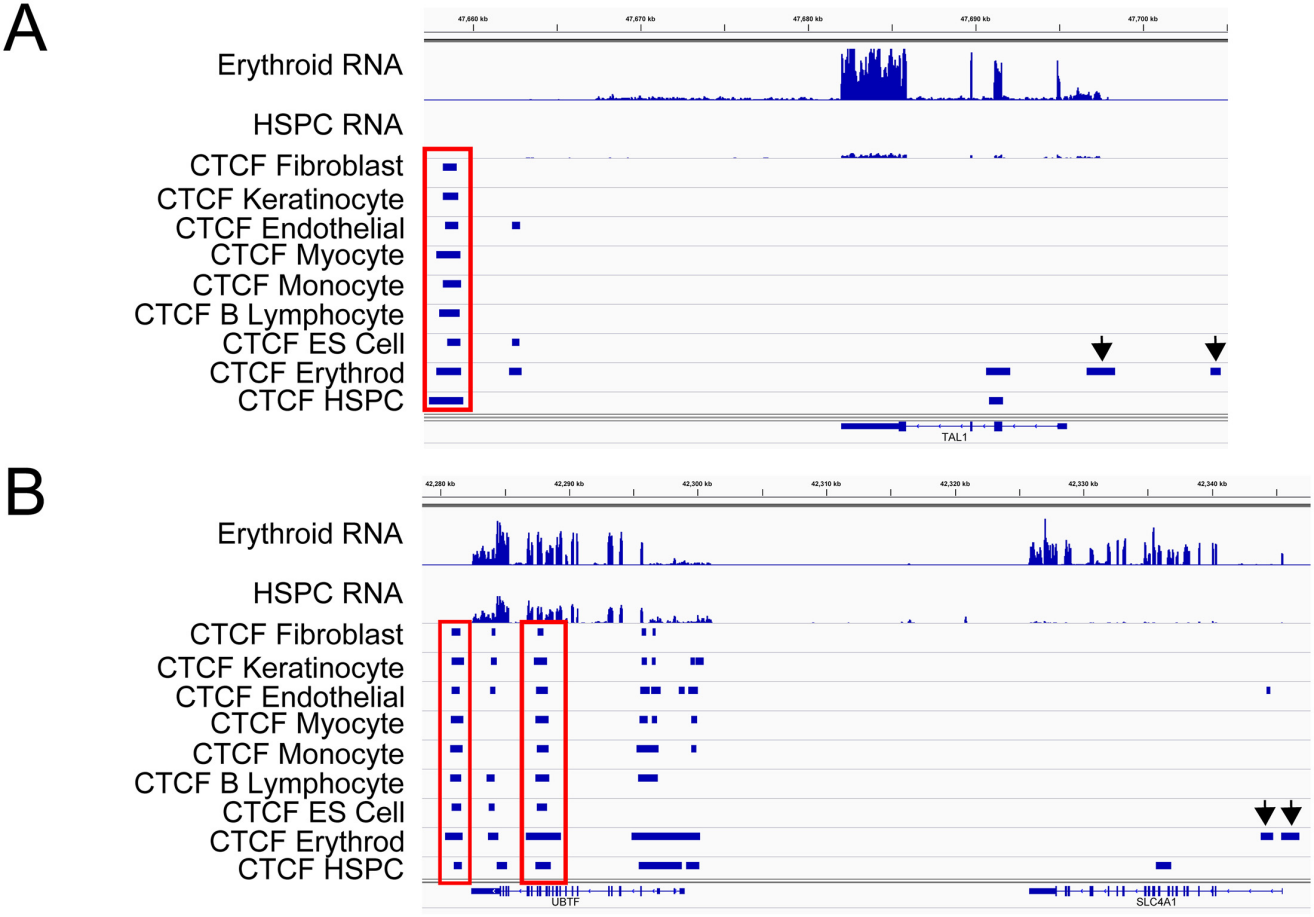
Invariant and cell type-specific CTFC sites. Patterns of CTCF occupancy in HSPC and erythroid cell chromatin were compared to CTCF occupancy in several human ENCODE ChIP-seq data sets, including fibroblast, keratinocyte, endothelial, myocyte, monocyte, lymphocyte, embryonic stem (ES) cell, erythroid and HSPC cells. **A.** At the *TAL1* locus, a 3’ site of invariant CTCF binding marked by the rectangle is present in all cell types. Two sites of erythroid-specific CTCF binding, denoted by the arrows, are present 5’ of the gene. Corresponding RNA-seq tracks in HSPC and erythroid cells are shown at the top. Genomic coordinates: Chr1:47,600–47,700. **B.** At the *UBTF* and *SLC4A1* loci, there are 2 sets of invariant CTCF binding, marked by rectangles, 3’ of the *UBTF* locus, present in all cell types. One site of erythroid-specific CTCF binding, denoted by the arrows, are present 5’ of the *SLC4A1* locus. Corresponding RNA-seq tracks in HSPC and erythroid cells are shown at the top. Genomic coordinates: Chr17:42,280–42,340.

CTCF has been reported to have sites of both cell-type specific and cell-type invariant binding, with ~40–60% of sites demonstrating cell-type specificity. Patterns of CTCF occupancy in HSPC and erythroid cell chromatin were compared to each other and to CTCF occupancy in several human ENCODE ChIP-seq data sets, including monocyte (CD14+), lymphoblastoid (G17828), embryonic stem cell (H1ES), human cardiac myocytes (HCM), human mammary fibroblasts (HMF), human umbilical vein endothelial (HUVEC), and normal human epidermal keratinocytes (NHEK) (Table 1). Cell type-specific CTCF sites were more common in HSPC cell chromatin, with 51% (25,912) of CTCF sites specific to HSPC cells, *i*.*e*. not present in any of the 7 ENCODE data sets. Twenty six percent (13,307) of the CTCF sites in HSPC cells were invariant, i.e. present in all 7 data sets compared to 39% (19,396) in erythroid cells. Typical cell-type specific and invariant CTCF binding sites are shown at several gene loci in erythroid cells (Fig 2).

**Table 1:** Comparison of CTCF binding sites in hematopoietic stem and progenitor cell (HSPC) and erythroid cell chromatin with CTCF binding in multiple cell types.

*Cell Type*	Data Source*	*Number of CTCF Binding Sites Identified*	*Number of Common CTCF Sites in HSPC Cells*	*Number of Common CTCF Sites in Erythroid Cells*
HSPC	This report	50,798	23,105 (47%)	
Erythroid	This report	49,417		23,183 (45%)
CD14	Broad	40,187	16,497 (32%)	24,867 (50%)
GM12878	Broad	37,570	19,064 (38%)	29,316 (59%)
H1ES	Broad	54,489	19,747 (39%)	29,898 (61%)
HCM	UWashington	44,259	19,799 (39%)	31,253 (63%)
HMF	UWashington	51,723	19,392 (38%)	31,512 (64%)
HUVEC	UWashington	66,858	22,240 (44%)	36,453 (74%)
NHEK	Broad	53,766	19,292 (38%)	31,723 (64%)
K562	UWashington	54,076	22,021 (43%)	34,091 (69%)

* ENCODE ChIP-seq data from multiple cell types obtained from the UCSC database were compared to binding sites in primary human HSPC progenitor cells and primary nucleated human erythroid cells. Percents are the fraction of common sites divided by the total number of HSPC or erythroid CTCF sites.

### Cell-type specific CTCF sites are near highly expressed genes in erythroid cells but not HSPCs

**Fig 3:**
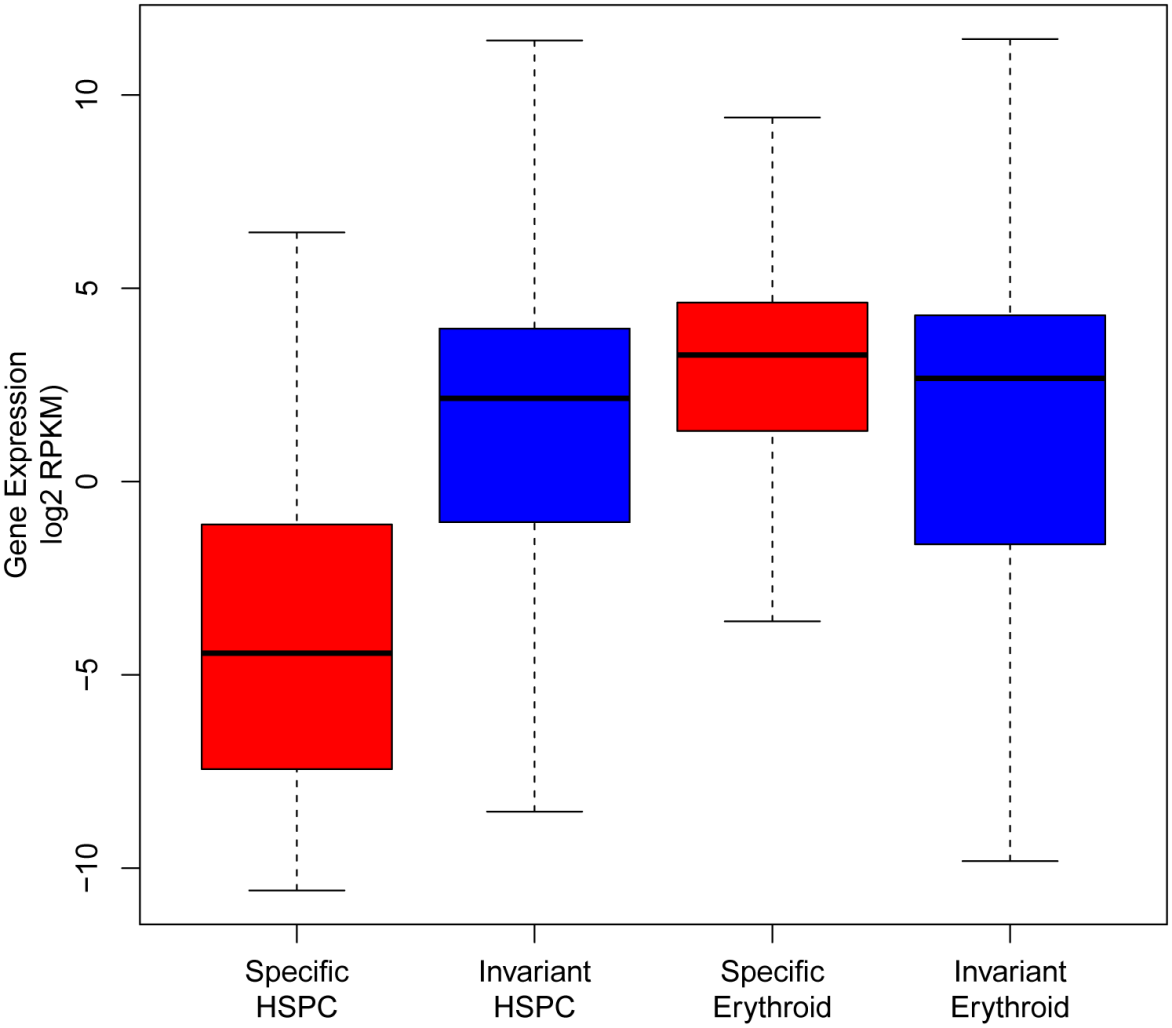
Gene expression and CTCF occupancy. Gene expression levels in primary human HSPC and erythroid cell mRNA were correlated with sites of CTCF occupancy by class within 1kb of the transcription start site.

Levels of mRNA expression were assessed in genes within 1kb of cell type-specific or invariant CTCF sites. Genes linked to erythroid-specific CTCF sites were expressed at significantly higher levels than those with invariant CTCF sites (*p*-value < 2.2e-16). In contrast, in HSPCs, genes linked to cell type-specific CTCF sites were expressed at significantly lower levels than genes linked to invariant CTCF sites (*p*-value < 2.2e-16)(Fig 3). A series of network and pathway analyses were performed on genes with cell-type specific CTCF binding sites.[36] Interestingly, genes within 1kb of erythroid cell-specific CTCF sites were highly significantly enriched for Gene Ontogeny Biological Process terms associated with hematopoiesis including “regulation of erythrocyte differentiation” and were enriched for Mouse Phenotype terms including “microcytic anemia” and “decreased mean corpuscular volume.”

### There is poor correlation of CTCF occupancy between primary erythroid cells and K562 cells

These studies were performed in primary human hematopoietic cells rather than in cells from transformed lines. K562 erythroleukemia cells have been utilized as a model of erythroid cell genetics and epigenetics by ENCODE. When comparing CTCF occupancy in human primary erythroid cells to K562 cells, only 69% of sites were shared (Table 1).

### Repressive chromatin domains increase in number during erythropoiesis

Cellular differentiation has been associated with reorganization and expansion of repressive chromatin domains in mammalian genome with silencing of the genes in the domain.[37–39] To examine repressive chromatin domains and their boundaries during hematopoiesis, ChIP-seq with an antibody against H3K27me3 as a marker of repressive chromatin was performed with HSPC and erythroid cells. Chromatin domains were identified using the Sicer program.[35] More H3K27me3 chromatin domains were identified in erythroid *vs*. HSPC cell chromatin (17,165 vs. 11,649, Table 2). In addition, average domain lengths were longer in erythroid compared to HSPC chromatin (12.2 vs. 8.3kb, Table 2), with the erythroid domains encompassing 6.7% of the genome compared to 3.1% in HSPC cells.

**Table 2:** Repressive chromatin domains and CTCF and cohesin^SA-1^occupancy at domain boundaries.

	HSPC cells	Erythroid Cells
Number of Domains	11649	17165
Average Domain Length	8.3 kb	12.2 kb
CTCF at Domain Boundaries	4832	3888
Cohesin^SA-1^ at Domain Boundaries	5093	3854
CTCF- Cohesin^SA-1^ co-localization at Domain Boundaries	2602	2180

Of the 17,165 H3K27me3 domains identified in erythroid cell chromatin, 59% (10,146) were specific to erythroid cells (*i*.*e*. not in CD34 cells). Thus a large number of tissue-specific repressive chromatin domains are found in differentiated erythroid cells. There was a strong anti-correlation of H3K27me3 domains with gene expression. This difference was much greater in erythroid cells than HSPCs (Figure H in S1 File).

### CTCF and cohesin^SA-1^ mark the boundaries of chromatin domains in a cell-type specific manner

In some cell types, CTCF has been observed to mark the boundaries of repressive chromatin domains in a cell-type specific manner.[40] To determine whether CTCF and cohesin^SA-1^ are present at domain boundaries in HSPC and erythroid cell chromatin, CTCF and cohesin^SA-1^ binding sites were mapped onto chromatin domains defined by H3K27me3 modification. Binding sites within 1 kb of a domain boundary were considered to mark the boundary of the domain.

**Fig 4:**
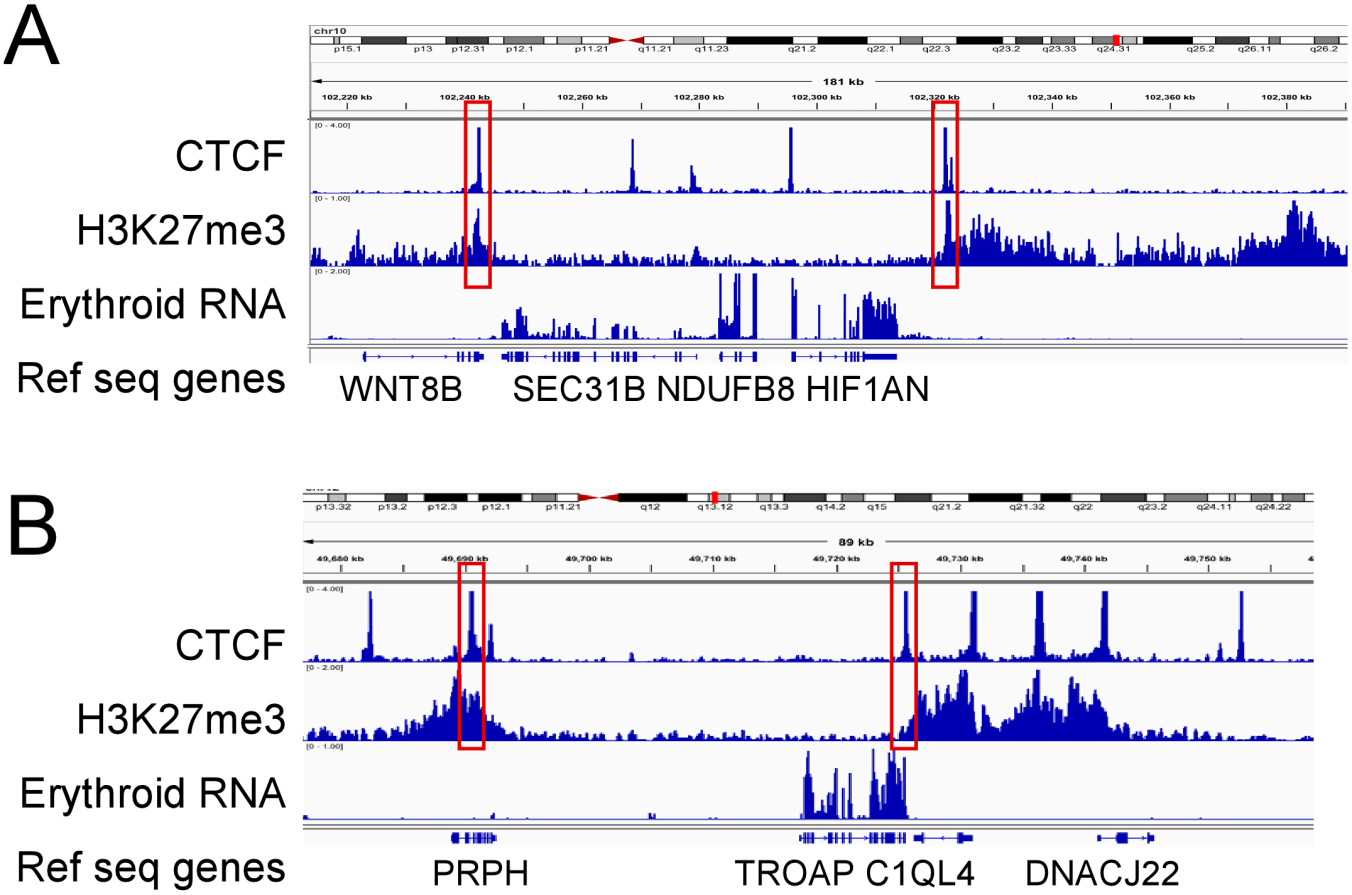
Repressive chromatin domains and CTCF occupancy. Representative integrated genome viewer (IGV) views of CTCF occupancy, repressive chromatin domains marked by H3K27me3 enrichment, and gene expression determined by RNA-seq in erythroid cells. **A.** Repressive chromatin domains marked by CTCF occupancy at their boundaries flank the *SEC31B*, *NDUFB8*, and *HIF1AN* genes. These 3 genes are expressed in erythroid cells, while the *WNT8B* gene, located in a repressive chromatin domain, is not. Genomic coordinates: Chr10:102,220–102,380. **B.** Repressive chromatin domains marked by CTCF occupancy at their boundaries flank the *TROAP and C1QL4* genes. These 2 genes are expressed in erythroid cells, while the flanking *PRPH* and *DNACJ22* genes, located in flanking repressive chromatin domains, are not. Genomic coordinates: Chr12:49,680–49,760.

There were 4,832 and 3,888 CTCF sites that marked domain boundaries in HSPC and erythroid cells, respectively (Table 2 and Fig 4). These CTCF sites were cell-type specific, as only 711 sites were shared between HSPCs and erythroid cells. Cohesin^SA-1^ was also found at domain boundaries, present at 5093 boundaries in HSPC cells and 3854 boundaries in erythroid cells.

**Fig 5:**
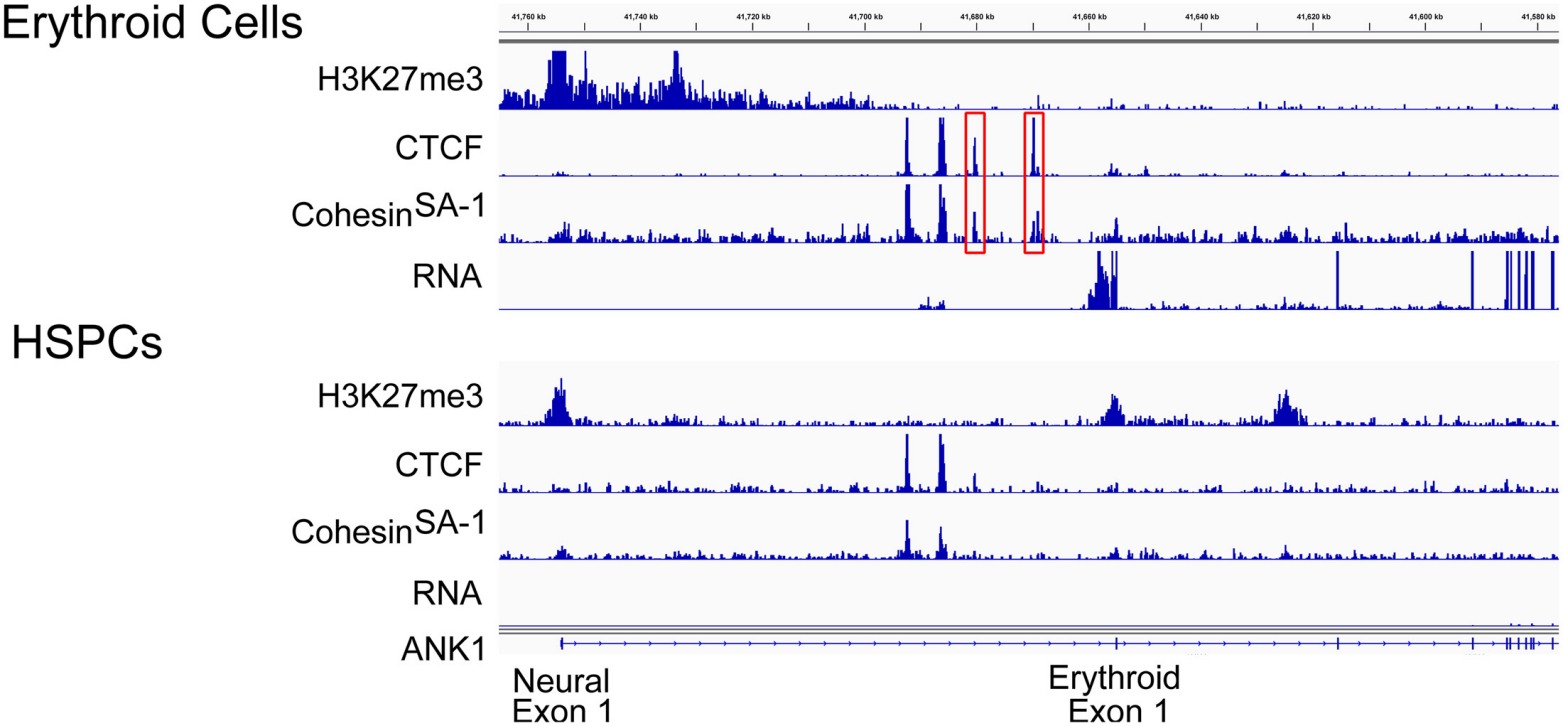
Repressive chromatin domains and CTCF-cohesin^SA-1^ co-occupancy. Representative integrated genome viewer (IGV) views of CTCF and cohesin^SA-1^ occupancy, repressive chromatin domains marked by H3K27me3 enrichment, and gene expression determined by RNA-seq in HSPC and erythroid cells. Multiple tissue-specific “exon 1s” are found at the 5’ end of the *ANK1* gene which all join in frame to exon 2, creating cDNA transcripts with unique 5’ ends. In erythroid cells (top), the sequence surrounding and including a neural-specific *ANK1* exon 1, located 5’ of the erythroid exon 1, is in a region of repressive chromatin, heavily modified by H3K27me3. At the boundary of this repressive chromatin domain are a pair of CTCF/cohesin^SA-1^ sites, present in erythroid but not HSPC chromatin, followed by the transcribed exons of the *ANK1* gene. *ANK1* is not expressed in HSPCs and this entire region is modified by H3K27 trimethylation (bottom). Genomic coordinates: Chr8:41,760–41,580.

CTCF frequently co-localized with cohesin^SA-1^ at domains, with 54% of CTCF sites at boundaries (*p*-value <0.001) and 56% of CTCF sites at boundaries (*p*-value <0.001) binding both proteins in HSPC and erythroid cell chromatin, respectively (Table 2). An example of CTCF and cohesin^SA-1^ at an erythroid-specific boundary is shown at the ankyrin-1 (*ANK1*) locus in Fig 5. Multiple tissue-specific “exon 1s” are found at the 5’ end of the *ANK1* gene which all join in frame to exon 2, creating cDNA transcripts with unique 5’ ends. In erythroid cells, the sequence surrounding and including a neural-specific *ANK1* exon 1, located 5’ of the erythroid exon 1, is in a region of repressive chromatin, heavily modified by H3K27me3 (Fig 5, top). At the boundary of this repressive chromatin domain are a pair of CTCF/cohesin^SA-1^ sites, present in erythroid but not HSPC chromatin, followed by the transcribed exons of the *ANK1* gene. *ANK1* is not expressed in HSPCs and this entire region is modified by H3K27 trimethylation (Fig 5, bottom). This region has been shown to functionally act as a barrier insulator *in vitro* and *in vivo*.[41] Together, these data indicate CTCF and cohesin^SA-1^ mark the boundaries of some repressive chromatin domains in a cell-type specific manner.

## Discussion

CTCF and cohesin^SA-1^ are distributed widely throughout the genomes of human HSPC hematopoietic stem and progenitor cells and differentiating erythroid cells. The finding of large numbers of co-occupied sites present at gene promoters in erythroid cells, not a common finding in all cell types studied to date, [42] is consistent with the recent observation that the cohesin complex is present at enhancers and active gene promoters.[8]

Although there were many shared sites of CTCF and cohesin^SA-1^ co-occupancy in both cell types, the majority of CTCF and cohesin^SA-1^ sites lacked the other protein. Similar to other highly differentiated cell types, cell-type specific CTCF sites were far more common in erythroid cell chromatin than HSPCs.

Detailed genome wide epigenetic studies have revealed a complex, higher order of chromosomal organization, with numerous, extensive chromatin domains. Repressive heterochromatin domains, defined by posttranslational histone modifications such as dimethylation of histone H3 lysine 9, trimethylation of histone H3 lysine 9, and trimethylation of histone H3 lysine 27, may extend over megabases in human cells.[37, 38] Studies comparing human embryonic stem cells to differentiated cell types have suggested that repressive chromatin domains increase in number and size with cellular differentiation, [37, 39] with silencing of the genes contained in the heterochromatin. In our studies, the number of H3K27me3 repressive chromatin domains doubled during erythroid development indicating that acquisition of repressive chromatin domains during erythropoiesis parallels embryonic stem cell development.

Our data indicate that many repressive chromatin domains in HSPC and erythroid cells have cell-type specific CTCF and cohesin^SA-1^ occupancy at their boundaries, suggesting that these proteins play a role in either domain establishment or maintenance. A subset of CTCF sites has been mapped to domain boundaries in T lymphocytes, HeLa cells, and Jurkat cells, leading to speculation that CTCF plays an important role in chromatin insulator function.[40] CTCF is not required for the barrier activity of the chicken HS4 insulator.[43] However, other reports have implicated a role for CTCF in barrier function, [40, 44] although this has not been supported by direct evidence.[45] Finally, it has been suggested that cohesin proteins may act as transcriptional insulators, [11] but again, studies providing direct evidence to support this hypothesis are lacking. Unraveling the numerous role(s) of CTCF and cohesin^SA-1^ at domain boundaries will provide considerable insight into our understanding of higher order chromatin structure and function. These genome wide datasets in human primary hematopoietic cells are excellent resources for future studies.

Much of the currently available data on chromatin architecture and transcription factor occupancy have been generated by ENCODE, which primarily utilized transformed cell lines for their studies. These studies were performed in primary human hematopoietic cells rather than in cells from transformed lines. K562 erythroleukemia cells, derived from a patient with chronic myelogenous leukemia in blast crisis and often used as surrogates for studies of erythroid gene function and regulation, have been utilized as a model of erythroid cell genetics and epigenetics by ENCODE. When comparing CTCF occupancy in human primary erythroid cells to K562 cells, only 69% of sites were shared. The lack of more extensive overlap may reflect developmental differences, as K562 cells are at significantly earlier stage of differentiation than R3/R4 erythroid cells, differences between primary cells and an immortalized cell line due to acquired aneuploidy, and/or other related changes acquired over time.[46]

Alterations in higher-order genome organization leading to perturbation in gene expression are being recognized as important mechanisms of inherited and acquired disease.[47] Because of their critical roles in organizing and maintaining higher order chromatin structure and regulating appropriate patterns of gene expression, perturbation of the structure or function of CTCF or cohesin^SA-1^ have been associated with disease phenotypes. Disruption or deletion of CTCF-associated insulators have been described in human disease such as loss of function of the DM1 insulator in myotonic dystrophy, and chromosomal deletions or translocations of regions containing CTCF binding sites in Beckwith-Wiedemann syndrome, Wilms’ tumor, and other various cancers.[44, 48] Perturbation of associated *cis*-sequences regulating their binding are another predicted mechanism of disease, [49, 50] as shown in a subset of cases of hereditary spherocytosis.[51]

Defects of the cohesin complex, collectively termed the “cohesinopathies” have been associated with several disorders with prominent developmental defects.[52] Roberts syndrome/SC-phocomelia and Cornelia de Lange syndrome patients suffer from mutations in cohesin complex-associated pathway proteins. Detailed analyses of these disorders indicate that distinct from its role in chromosome segregation, abnormalities of the cohesin network that alter gene expression and genome organization may underlie cohesinopathies.[53] Synthesis of data from detailed patient genetic studies and from functional genomics studies, such as these hematopoietic cell data sets, which identify regions of DNA with regulatory potential throughout the genome, will provide critical insight into our understanding of the complex mechanisms of genetic variation in inherited and acquired disease.

## Conclusions

Sites of CTCF and cohesin^SA-1^ occupancy, associated chromatin architecture, and related gene expression changed during erythropoiesis. Repressive chromatin domains increased in both number and size during hematopoiesis, with many more repressive domains in erythroid cells than HSPCs, with CTCF and cohesin^SA-1^ marking the boundaries of these repressive chromatin domains in a cell-type specific manner. These genomic data support the hypothesis that CTCF and cohesin^SA-1^ have multiple roles in the regulation of gene expression during erythropoiesis. Obtained from primary human erythroid cells, these datasets provide an important resource for studies of normal and perturbed erythropoiesis.

## Supporting Information

S1 File Supporting Figures. (Figure A) Analytic flow activated cell sorting analyses of cultured human primary erythroid cells. (Figure B) Correlation heat map using affinity (read count) data. (Figure C) Principal components analysis of affinity (read count) data. (Figure D) Heat map of affinity (read count) data for individual sites in individual ChIP-seq samples. (Figure E) Sites of CTCF and cohesin^SA-1^ occupancy in HSPCs and erythroid cells. (Figure F) Motif analysis using the Homer algorithm in HSPC. (Figure G). Motif analysis using the Homer algorithm in erythroid cells. (Figure H) Correlation of repressive domains and gene expression in HSPCs and erythroid cells.
(PDF)

S2 File Supporting Tables. (Table A) PCR primers for CTCF and cohesin^SA-1^ validation. (Table B) Read Count, Duplication and Strand Cross Correlation Analyses. (Table C) Quantitative ChIP Validation of CTCF binding Sites. (Table D) Quantitative ChIP Validation of cohesin^SA-1^ binding sites. (Table E) Summary of ChIP seq results.
(DOCX)
