# Supplementary material for: CTCF and CohesinSA-1 Mark Active Promoters and Boundaries of Repressive Chromatin Domains in Primary Human Erythroid Cells
Source: PLoS One. 2016 May 24;11(5):e0155378. doi: 10.1371/journal.pone.0155378 (PMC4878738; doi:10.1371/journal.pone.0155378)

SUPPLEMENTAL FIGURE A. *Analytic flow activated cell sorting analyses of cultured human primary erythroid cells.* Erythroid progenitor cells were cultured and isolated as described. Immunomagnetic bead selection was used to select a population of cells based on expression of CD71 (transferrin receptor) and CD235a (glycophorin A), representing the R3/R4 cell population of nucleated erythroid cells at >95% purity as assessed by analytic FACS.

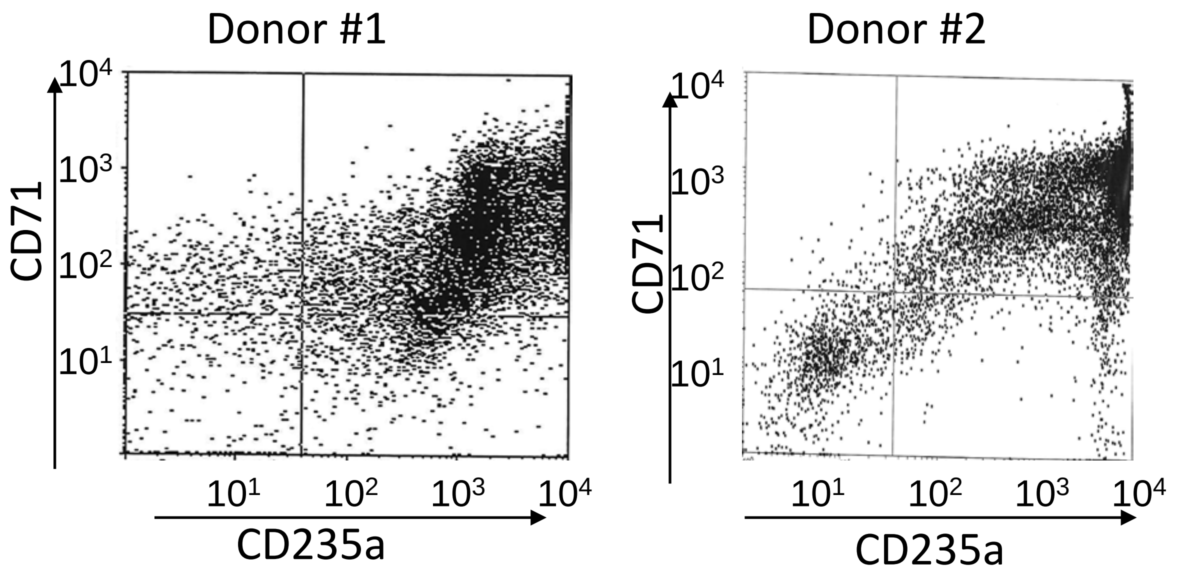

SUPPLEMENTAL FIGURE B. *Correlation heat map using affinity (read count) data*. Read counts of peak regions were obtained for all samples using the Diffbind package. Correlation values for all regions are displayed as a heat map where the amount of correlation is as displayed in the key. Patterns of similarity are revealed by hierarchical clustering.

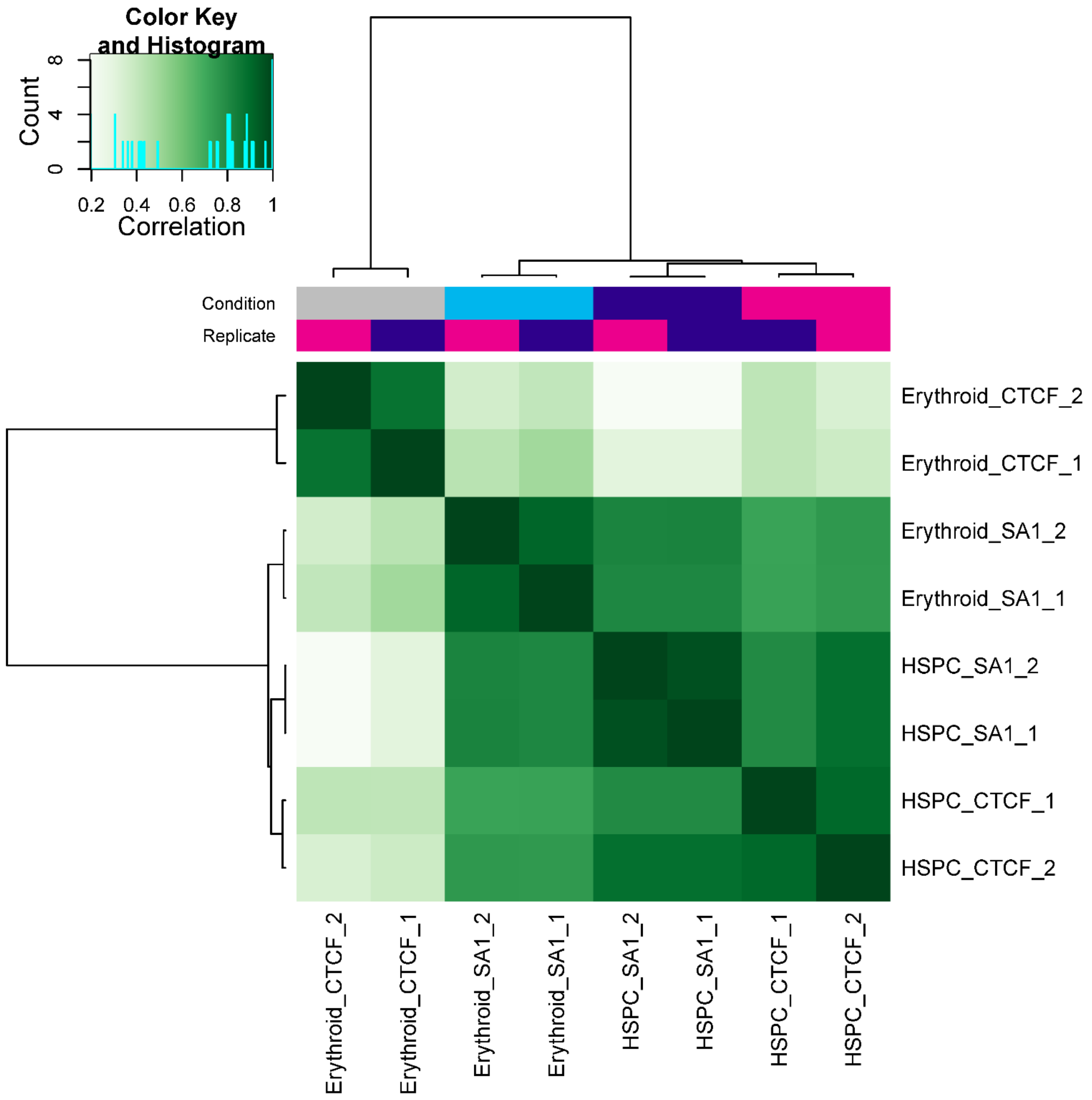

SUPPLEMENTAL FIGURE C. *Principal components analysis of affinity (read count) data*. Read counts of peak regions were obtained for all samples using the Diffbind package. The data is subjected to Principal Components analysis to reveal the patterns of sample relatedness.

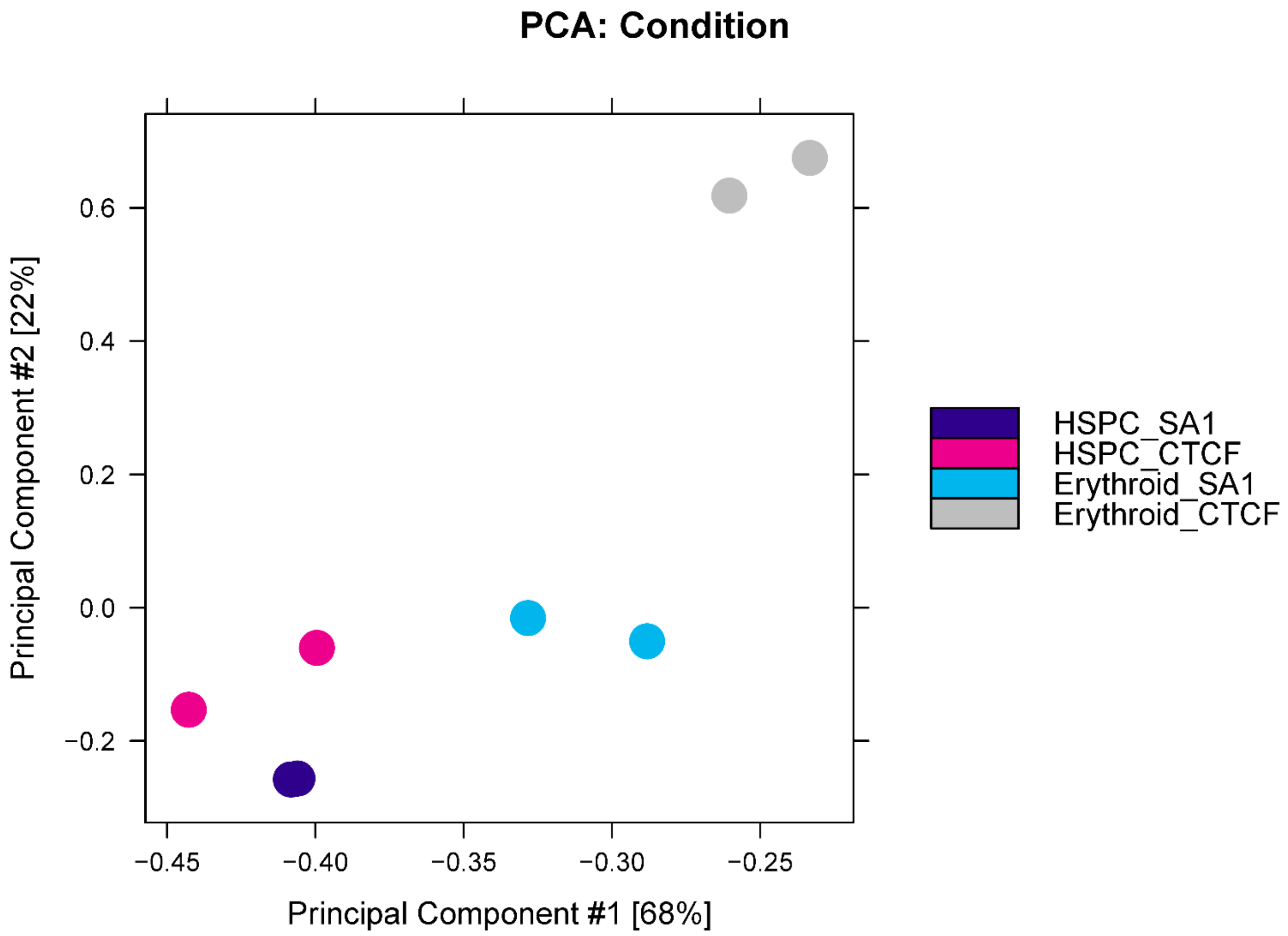

SUPPLEMENTAL FIGURE D. *Heat map of affinity (read count) data for individual sites in individual ChIP-seq samples*. Read counts of peak regions were obtained for all samples using the Diffbind package. Normalized binding values for all regions are displayed as a heat map where the amount of binding is as displayed in the key. Patterns of similarity are revealed by hierarchical clustering.

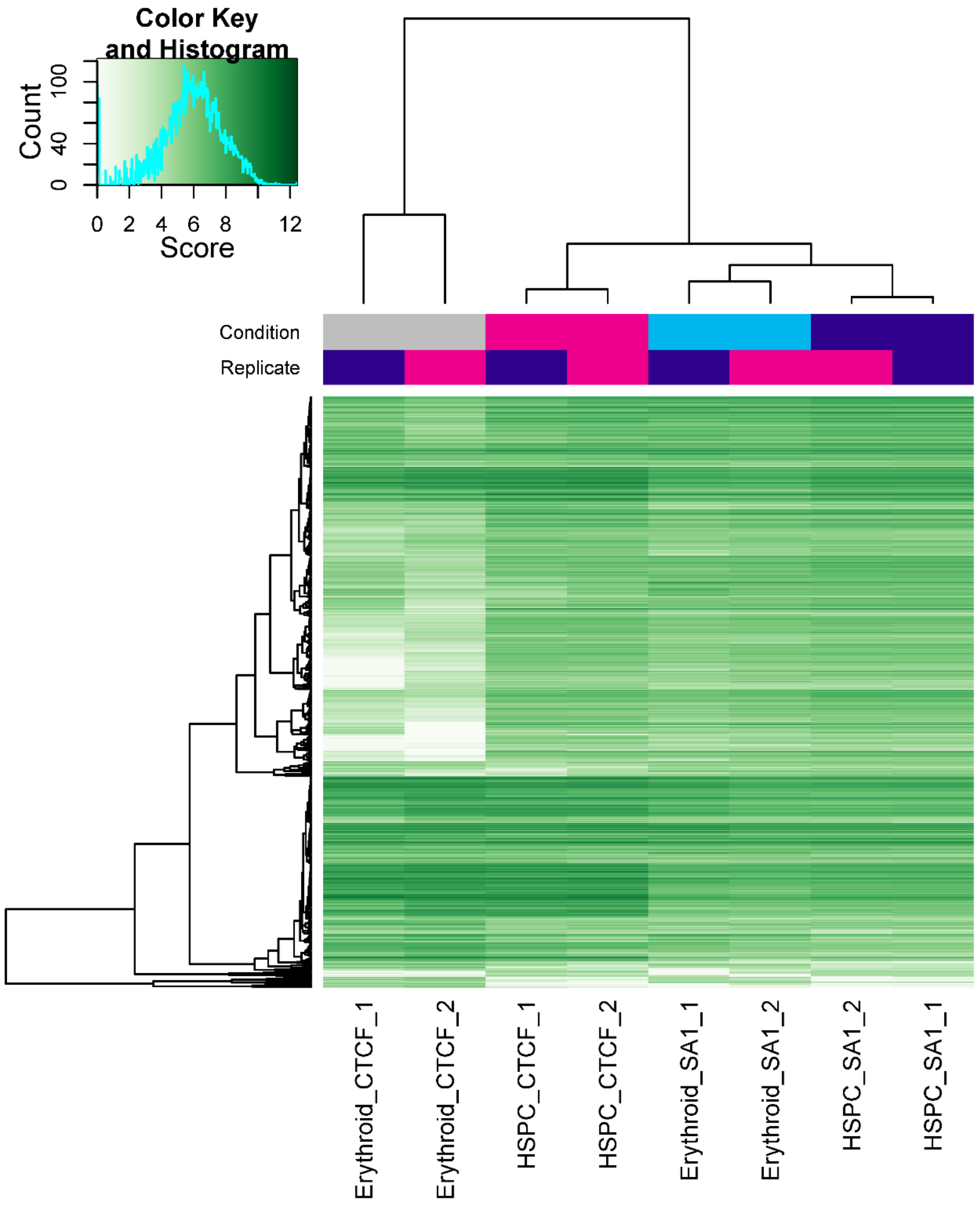

SUPPLEMENTAL FIGURE E. *Sites of CTCF and cohesin$^{SA\text{-}1}$ occupancy in HSPCs and erythroid cells.* Four-way Venn diagram representation of overlapping and unique sites of CTCF and cohesin$^{SA\text{-}1}$ occupancy in HSPC and erythroid cells.

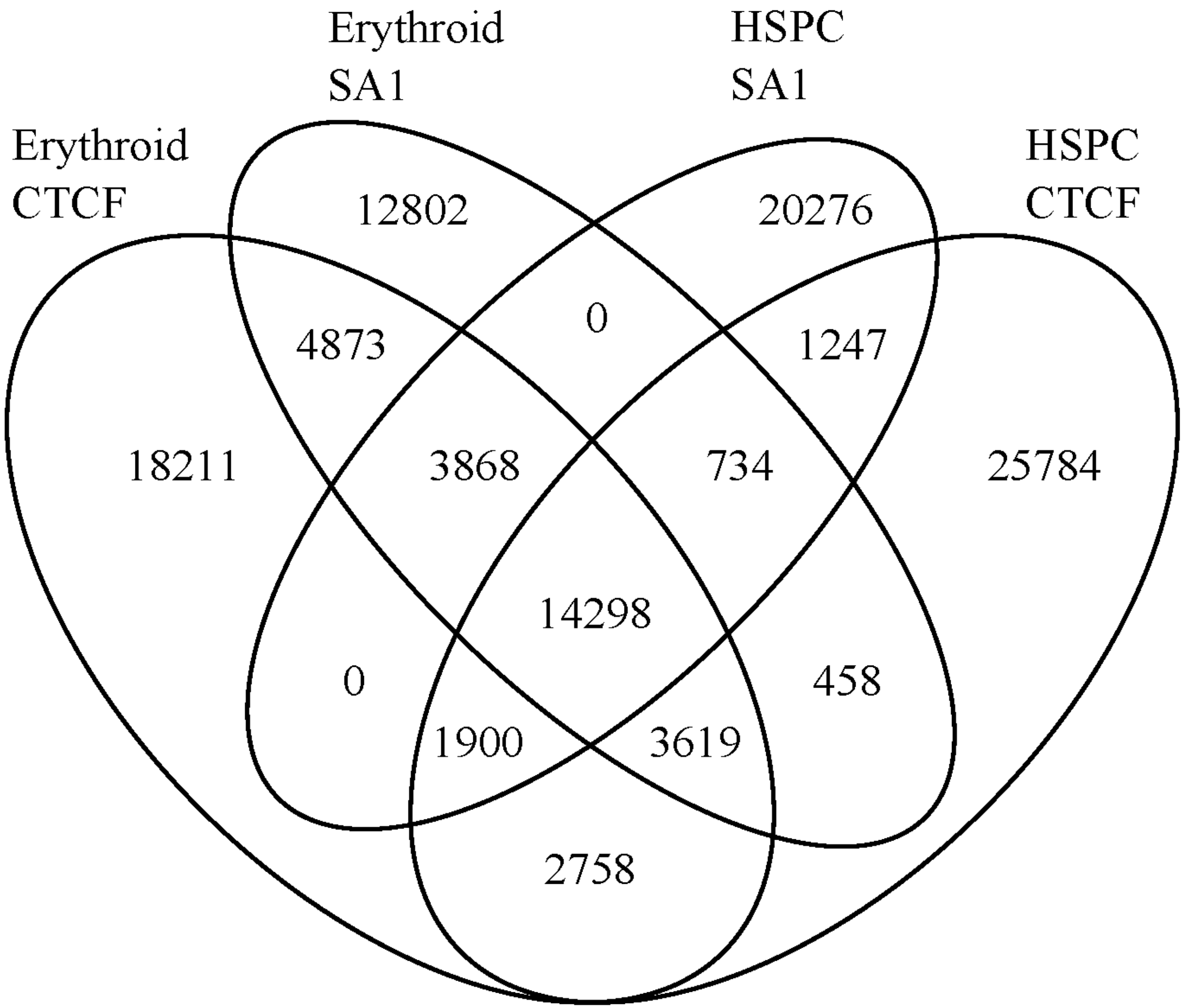

SUPPLEMENTAL FIGURE F. *Motif analysis using the Homer algorithm*. The top regulatory protein binding sites identified by the Homer algorithm searching ± 50bp from summits of at sites of CTCF and cohesin^{SA-1} binding. Hematopoietic stem and progenitor cells. The top four motifs ranked by *p*-value are shown.

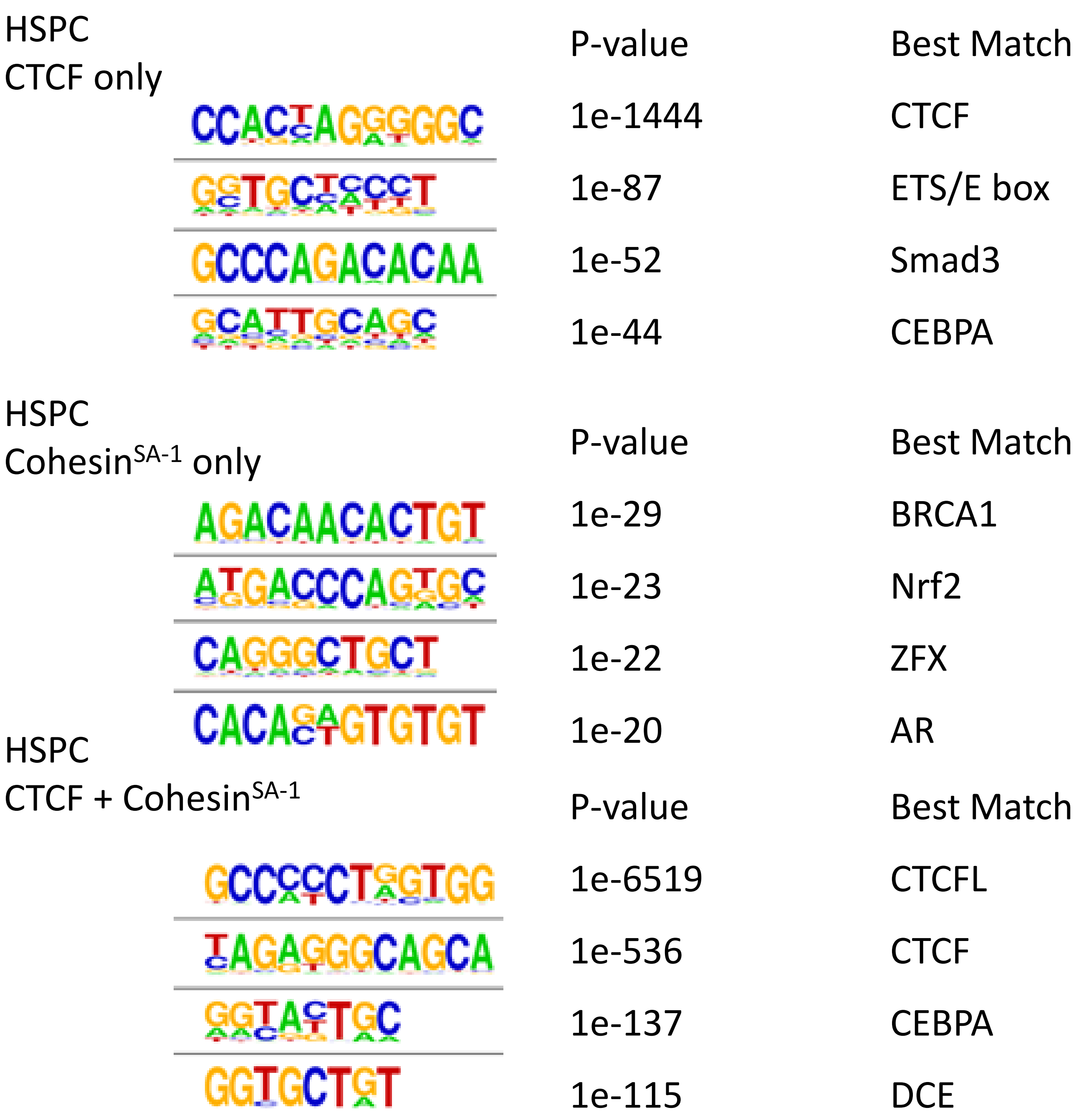

SUPPLEMENTAL FIGURE G. *Motif analysis using the Homer algorithm*. The top regulatory protein binding sites identified by the Homer algorithm searching ± 50bp from summits of at sites of CTCF and cohesin$^{SA\text{-}1}$ binding. Erythroid cells. The top four motifs ranked by *p*-value are shown.

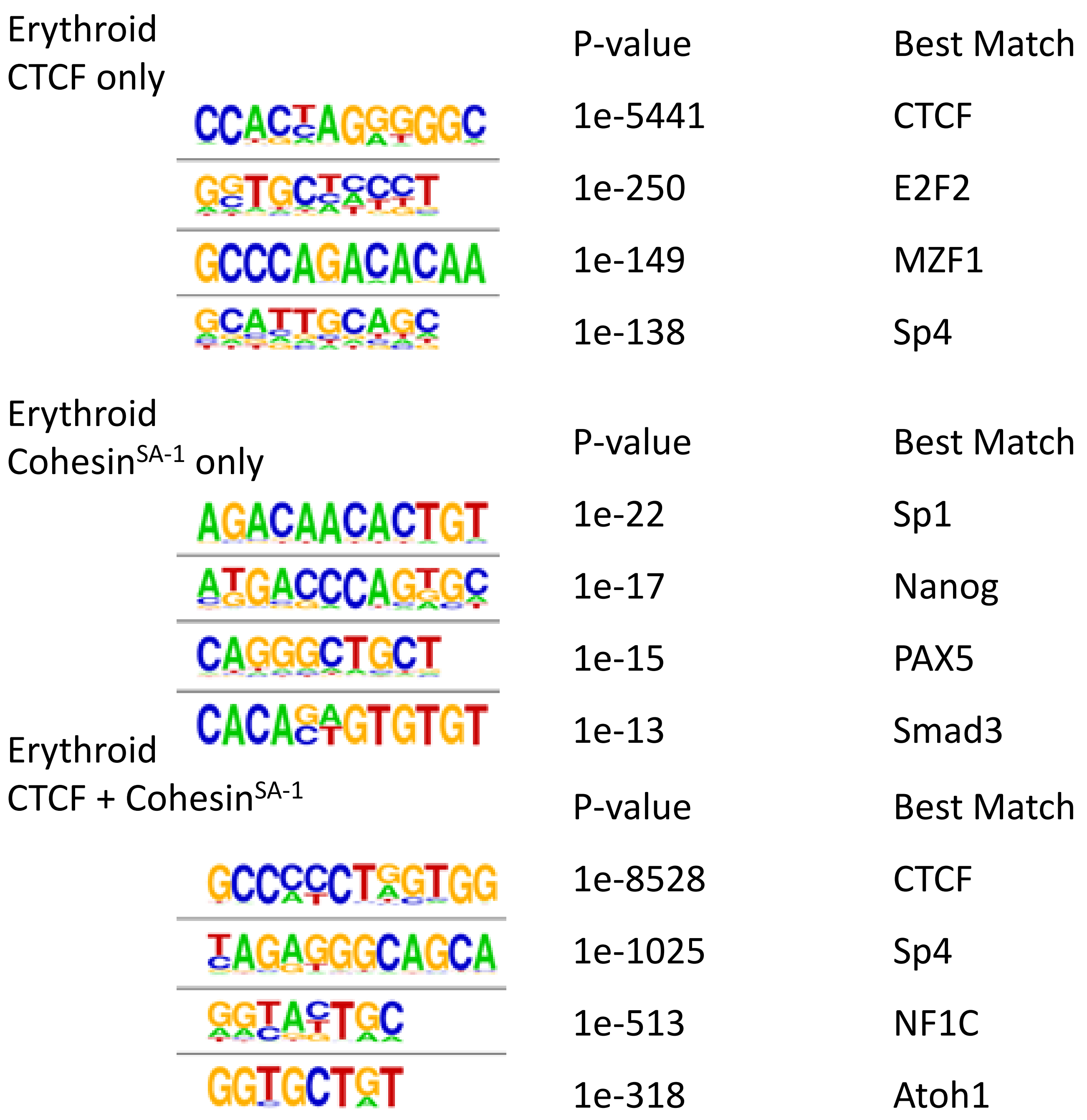

SUPPLEMENTAL FIGURE H. *Correlation of repressive domains and gene expression in HSPCs and erythroid cells*. Gene expression levels (log2 RPKM) in primary human HSPC and erythroid cell mRNA for genes with or without repressive chromatin domains marked H3K27me3 at their promoters are displayed. There was a strong anti-correlation of gene expression with H3K27me3 domains.

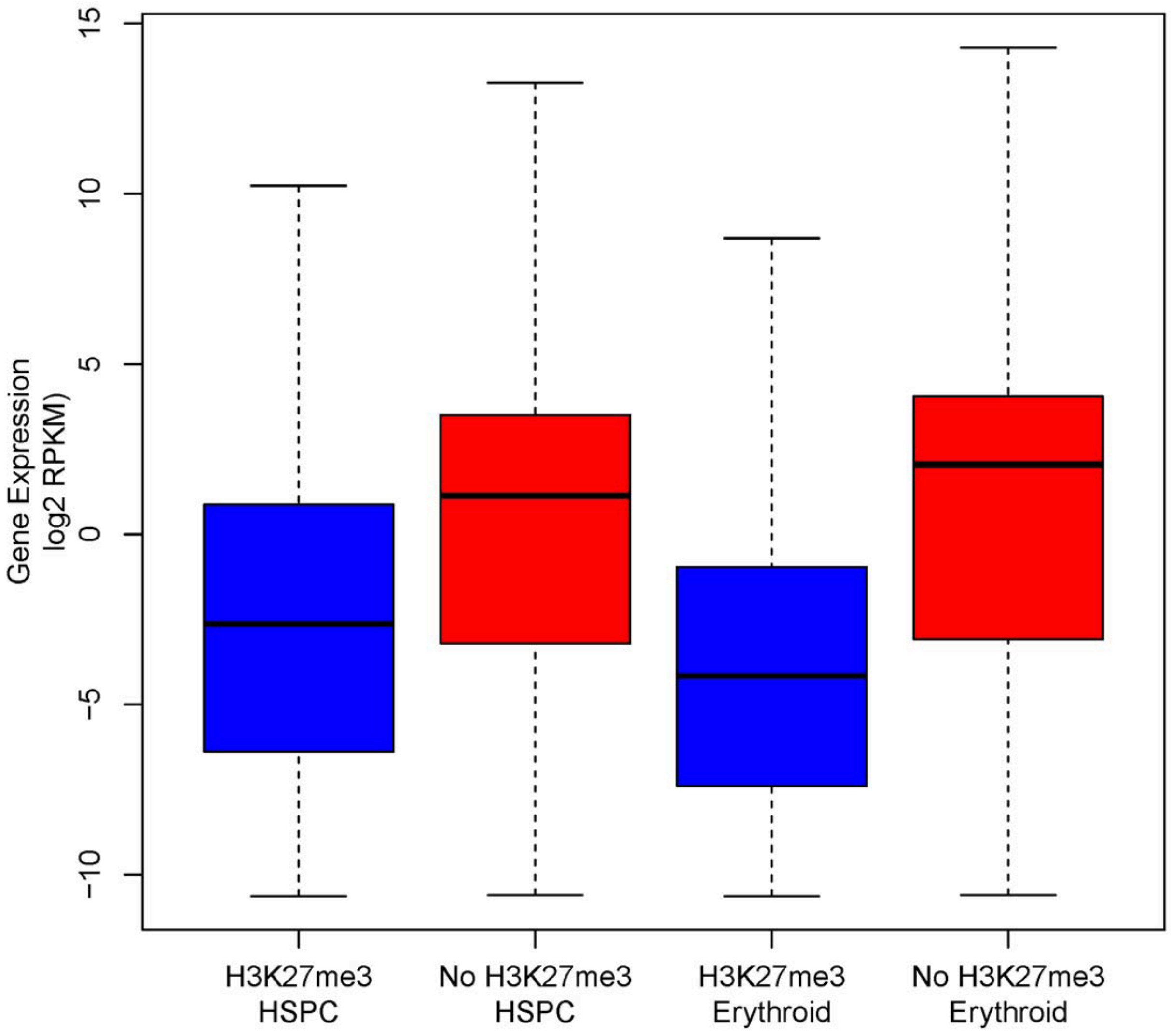

Supplement: S1 File — (Figure A) Analytic flow activated cell sorting analyses of cultured human primary erythroid cells. (Figure B) Correlation heat map using affinity (read count) data. (Figure C) Principal components analysis of affinity (read count) data. (Figure D) Heat map of affinity (read count) data for individual sites in individual ChIP-seq samples. (Figure E) Sites of CTCF and cohesinSA-1 occupancy in HSPCs and erythroid cells. (Figure F) Motif analysis using the Homer algorithm in HSPC. (Figure G). Motif analysis using the Homer algorithm in erythroid cells. (Figure H) Correlation of repressive domains and gene expression in HSPCs and erythroid cells. (PDF) [file pone.0155378.s001.pdf]
