# Supplementary material for: CTCF and CohesinSA-1 Mark Active Promoters and Boundaries of Repressive Chromatin Domains in Primary Human Erythroid Cells
Source: PLoS One. 2016 May 24;11(5):e0155378. doi: 10.1371/journal.pone.0155378 (PMC4878738; doi:10.1371/journal.pone.0155378)
Supplement: S2 File — (Table A) PCR primers for CTCF and cohesinSA-1 validation. (Table B) Read Count, Duplication and Strand Cross Correlation Analyses. (Table C) Quantitative ChIP Validation of CTCF binding Sites. (Table D) Quantitative ChIP Validation of cohesinSA-1 binding sites. (Table E) Summary of ChIP seq results. (DOCX) [file pone.0155378.s002.docx]

**SUPPLEMENTAL DATA**

CTCF and Cohesin^SA-1^ Mark the Active Promoters and Boundaries of Repressive Chromatin Domains in Erythropoiesis

Laurie A Steiner, Vincent Schulz, Yelena Makismova, Kimberly Lezon-Geyda and Patrick G Gallagher

Supplemental Table A: PCR primers for CTCF and cohesin^SA-1^ validation

Supplemental Table B: Read Count, Duplication and Strand Cross Correlation Analyses

Supplemental Table C: Quantitative ChIP Validation of CTCF binding Sites

Supplemental Table D: Quantitative ChIP Validation of cohesin^SA-1^ binding sites

Supplemental Table E: Summary of ChIP seq results

Supplemental Table A: PCR Primers for Quantitative ChIP Validation of CTCF and Cohesin^SA-1^ Binding Sites

| *ANK1* | intron 1B | Forward | GCAATCTCTGCTGTCCCATT |
| --- | --- | --- | --- |
|  |  | Reverse | AGCTTCTGAAAGCCGAGTCA |
| *DMTN/EPB49* | alt promoter | Forward | TCTCCCCAGCAACCACTTAC |
|  |  | Reverse | GAGAGGGGTACTCACCACGA |
| *EPB41* | 3' flank | Forward | GTTCAATGCAGGAGCCAGAC |
|  |  | Reverse | CTGCCTTGCTCAACAAGCTAC |
| *HBQ1* | coding | Forward | AAGTTCCTGAGCCACGTTATCT |
|  |  | Reverse | TTACTCAAACACGGGGAAGG |
| *RUNX1* | promoter | Forward | AGCGTTCGAGGATAAAAGACA |
|  |  | Reverse | GTCCGAGAGTCCCCACCT |
| *STK19* | coding | Forward | CGAAGGATGCAAAAGTGGTT |
|  |  | Reverse | CCTTCGTGAAGCTCACACCT |
| ENCODE (+) Control#1 | Chr. 3 | Forward | CCGCTGTGACTGGCTAA |
|  |  | Reverse | ATGTAATGACCACCCTGCCC |
| ENCODE (+) Control#2 | Chr. 9 | Forward | CAGGGGGCAAGAGTGATCC |
|  |  | Reverse | CTAAAGGGTGTCACCTGGG |
| Negative Control #1 | Chr. 17 | Forward | GGCTTCAACCGGACTGTGCGT |
|  |  | Reverse | TGTGGCTCGGACCTCGCAGT |
| Negative Control #2 | Chr. 1 | Forward | AAAGTCACCCGCCTTTCC |
|  |  | Reverse | CGAGGTCATGGAGCAGTT |

Supplemental Table B: Read Count, Duplication and Strand Cross Correlation Analyses

| **Cell Type** | **Factor** | **Replicate** | **Total Reads** | **Mapped Reads** | **Uniquely Mapped** | **Duplicated** | **RSC** | **Quality Tag** | **# Filtered Peaks** |
| --- | --- | --- | --- | --- | --- | --- | --- | --- | --- |
| HSPC | SA1 | 1 | 33,635,225 | 31,514,783 | 29,511,782 | 6.56% | 1.755254 | 2 | 42072 |
| HSPC | CTCF | 1 | 24,526,806 | 21,613,686 | 20,042,374 | 4.84% | 1.490195 | 1 | 50798 |
| Erythroid | SA1 | 1 | 36,805,676 | 34,744,156 | 32,533,738 | 4.58% | 1.804536 | 2 | 40511 |
| Erythroid | CTCF | 1 | 16,503,251 | 16,009,394 | 14,870,518 | 8.06% | 2.170706 | 2 | 49417 |
| HSPC | SA1 | 2 | 32,770,211 | 31,012,793 | 29,043,207 | 7.07% | 1.766919 | 2 | 41680 |
| HSPC | CTCF | 2 | 31,137,673 | 27,731,594 | 25,731,126 | 9.26% | 1.978105 | 2 | 41234 |
| Erythroid | SA1 | 2 | 43,332,116 | 41,322,749 | 38,648,746 | 3.24% | 1.485462 | 1 | 15355 |
| Erythroid | CTCF | 2 | 26,258,995 | 24,197,588 | 22,632,106 | 9.37% | 1.404095 | 1 | 39913 |

| Total Reads: total number of reads including unmapped, mapped and duplicate |
| --- |
| Mapped Reads: Number of sequence reads that map to hg19 human genome sequence |
| Uniquely Mapped: Number of sequence reads that map to only one location in hg19 human genome sequence |
| Duplicated: % reads that have identical mapping location to another read. |
| RSC: Relative strand cross-correlation coefficient. The RSC is the ratio of the fragment-length cross-correlation value minus the background cross-correlation value, divided by the phantom-peak cross-correlation value minus the background cross-correlation value. The minimum possible value is 0 (no signal), highly enriched experiments have values greater than 1, and values much less than 1 may indicate low quality. |
| QualityTag: Quality tag based on thresholded RSC (codes: -2:veryLow,-1:Low,0:Medium,1:High,2:veryHigh) |
| # Filtered Peaks: Number of MACS called peaks above fold change threshold |

Supplemental Table C. Quantitative ChIP Validation of CTCF Binding Sites

|  | | |  |  |  |  |
| --- | --- | --- | --- | --- | --- | --- |
|  | | |  |  |  |  |
| Gene | | |  | Enrichment* |  | Enrichment* |
|  | | |  | HSPC |  | Erythroid |
| *ANK1* | | |  | 8.28+0.94 |  | 37.14+0.96 |
| *RUNX1* | | |  | 2.65+0.37 |  | 49.18+0.96 |
| *EPB41* | | |  | 2.05+0.91 |  | 12.47+0.69 |
| *DMTN/EPB49* | | |  | 8.18+0.91 |  | 36.59+0.94 |
| ENCODE (+) Control#1 | | | | 7.18+0.96 |  | 8.55+0.96 |
| Negative Control #1 | | |  | 0.51+0.98 |  | 1.33+0.95 |
| Negative Control #2 | | |  | 2.05+0.98 |  | 0.52+0.76 |
|  | | |  |  |  |  |
| *Enrichment is relative to total input. | | | | |  |  |
|  |  |  | | |  |  |

Supplemental Table D: Quantitative ChIP Validation of Cohesin^SA-1^ Binding Sites

|  |  |  |  | |  | |
| --- | --- | --- | --- | --- | --- | --- |
|  |  |  |  | |  | |
| Gene |  | Enrichment* |  | | Enrichment* | |
|  |  | HSPC |  | | Erythroid | |
| *STK19* |  | 3.22+0.96 |  | | 2.12+0.95 | |
| Chr 9 |  | 4.31+0.90 |  | | 5.05+0.96 | |
| *HBQ1* |  | 5.65+0.92 |  | | 14.83+0.94 | |
| Negative Control #1 |  | 1.04+0.93 |  | | 0.96+0.98 | |
| Negative Control #2 |  | 1.32+0.78 |  | | 1.32+0.82 | |
|  |  |  |  | |  | |
| *Enrichment is relative to total input. | | |  |  | |  |

Supplemental Table E. Summary of ChIP seq Results

| **Sample** | **Total Reads** | **Uniquely Mapped Reads** | **Non-Duplicated Number of Peaks**  **Reads** | |  |
| --- | --- | --- | --- | --- | --- |
| HSPC CTCF | 24,526,806 | 20,042,374 | 19,142,773 | 50,798 | |
| HSPC SA-1 | 33,635,225 | 29,511,782 | 27,633,310 | 42,072 | |
| HSPC H3K27me3 | 16,226,959 | 13,897,861 | 13,550,969 |  | |
| HSPC Total Input | 30,897,713 | 27,902,244 | 27,362,695 |  | |
| Erythroid CTCF | 16,503,251 | 14,870,518 | 13,650,771 | 49,417 | |
| Erythroid SA-1 | 36,805,676 | 32,533,738 | 31,179,932 | 40,511 | |
| Erythroid H3K27me3 | 25,298,172 | 21,478,777 | 20,884,278 |  | |
| Erythroid Total Input | 32,937,093 | 29,395,325 | 28,870,106 |  | |
